# Systems Level Analysis and Identification of Pathways and Key Genes Associated with Delirium

**DOI:** 10.3390/genes11101225

**Published:** 2020-10-19

**Authors:** Yukiko Takahashi, Tomoyoshi Terada, Yoshinori Muto

**Affiliations:** 1 United Graduate School of Drug Discovery and Medical Information Sciences, Gifu University, 1-1, Yanagido, Gifu 501-1194, Japan; yu-taka@gifu-u.ac.jp (Y.T.); tterada@gifu-u.ac.jp (T.T.); 2 Department of Adult Nursing (Acute phase), Gifu University School of Medicine, 1-1, Yanagido, Gifu 501-1193, Japan; 3 Department of Functional Bioscience, Gifu University School of Medicine, 1-1, Yanagido, Gifu 501-1193, Japan

**Keywords:** delirium, protein–protein interaction, network, human accelerated regions, APP

## Abstract

Delirium is a complex pathophysiological process, and multiple contributing mechanisms have been identified. However, it is largely unclear how the genes associated with delirium contribute and which of them play key roles. In this study, the genes associated with delirium were retrieved from the Comparative Toxicogenomics Database (CTD) and integrated through a protein–protein interaction (PPI) network. Delirium-associated genes formed a highly interconnected PPI subnetwork, indicating a high tendency to interact and agglomerate. Using the Molecular Complex Detection (MCODE) algorithm, we identified the top two delirium-relevant network modules, M1 and M5, that have the most significant enrichments for the delirium-related gene sets. Functional enrichment analysis showed that genes related to neurotransmitter receptor activity were enriched in both modules. Moreover, analyses with genes located in human accelerated regions (HARs) provided evidence that HAR-Brain genes were overrepresented in the delirium-relevant network modules. We found that four of the HAR-Brain genes, namely APP, PLCB1, NPY, and HTR2A, in the M1 module were highly connected and appeared to exhibit hub properties, which might play vital roles in delirium development. Further understanding of the function of the identified modules and member genes could help to identify therapeutic intervention targets and diagnostic biomarkers for delirium.

## 1. Introduction

Delirium is characterized by fluctuating disturbances in attention, cognition, and self-awareness, which can arise acutely, either in the absence of prior intellectual impairment or superimposed on chronic intellectual impairment [1,2,3]. Delirium is considered the most common complication afflicting hospitalized elderly patients, and is associated with poor outcomes, including increased hospital stays and high morbidity and mortality rates [4]. The pathophysiological underpinnings of delirium are complex, and multiple contributing mechanisms have been identified [5,6]. These include direct and indirect effects of inflammatory, neuroendocrine, and gross anatomic or neurodegenerative mechanisms [7]. Moreover, candidate gene association studies on delirium have shown that apolipoprotein E [8], catechol-O-methyltransferase (COMT) [9], and dopamine-signaling genes such as dopamine receptor 2 [10] are related to the risk of delirium. Interleukin-related genes and the sodium/hydrogen exchanger gene were also identified as risk factors for delirium by a delirium-focused genome-wide association study (GWAS) [6]. Although these factors are thought to be important for the pathogenesis of delirium, it is largely unclear how the genes associated with delirium contribute and which of them play key roles. Other possible mechanisms for the pathogenesis might also need to be considered.

Many chemicals induce delirium and may be considered the most simple causative agents [11]. Certain classes of psychoactive drugs, such as narcotics and anticholinergic medications, more commonly cause delirium [12]. These agents induce delirium by disrupting brain states, but genetic differences could also increase susceptibility to the mechanisms of delirium by anticholinergic and other drugs. During the last decade, a wealth of data on the association between chemicals, genes, and diseases has become available from various toxicogenomic studies. The Comparative Toxicogenomics Database (CTD) is a public repository that aims to analyze the impact of chemicals on human health [13]. The CTD provides information on chemical–gene interactions, chemical–disease, and gene–disease relationships, to assist those investigating the mechanisms of diseases due to chemical factors. There are thousands of curated genes associated with delirium in the CTD that are available for use as candidate genes to be prioritized in relation to delirium. The rapid development of omics and computational technologies has provided the frameworks for network medicine, which combines principles and approaches of systems biology and network science [14]. Network approach is often used to elucidate the molecular mechanism of diseases and providing extensive knowledge on the genes and their interaction that can be used to improve diagnosis and prevention of the diseases [15,16]. Therefore, we considered that it would be useful to collect all the delirium-gene data from the CTD and apply them to a network-based analysis, to identify new key genes.

In the present study, the genes associated with delirium were retrieved from the CTD to identify molecular pathways and key genes involved in the pathogenesis of delirium. We performed a system-level analysis by integrating protein−protein interaction (PPI) network analysis, sub-network module analysis, and GO and KEGG pathway enrichment analysis, and also referred to evolutionary data for human accelerated regions (HARs). We found that the delirium-associated genes obtained from the CTD encoded a significantly connected molecular network. Sub-network module analysis identified two highly connected network modules, which were enriched for genes related to neurological functions and delirium risk.

## 2. Materials and Methods 

### 2.1. Genes Associated with Delirium

The Comparative Toxicogenomics Database (CTD, http://ctdbase.org/) provides information on chemical–gene/protein interactions and chemical–disease and gene–disease relationships, to facilitate researchers investigating the mechanisms of diseases caused by chemicals or chemical-related genes [13]. Delirium-associated gene data were obtained from the CTD by searching for genes by using the keyword “delirium” on 8 February 2020. In order to rank the genes associated with delirium, we sorted the list by the CTD inference score. The top-ranking genes with scores > 40 were taken as the seed genes in this study.

### 2.2. Construction of a Human Protein−Protein Interaction (PPI) Network

Protein-interaction data were downloaded from the iRefIndex database (http://irefindex.org) as an initial dataset. iRefIndex is a union of the many primary PPI databases, including BIND, BioGRID, CORUM, DIP, HPRD, InnateDB, IntAct, MatrixDB, MINT, MPact, MPIDB, and MPPI [17]. To increase the confidence and the completeness of the PPI network, we integrated PPIs from the large-scale BioPlex 2.0 interaction dataset [18] obtained by high-throughput affinity purification mass spectrometry. In addition, we incorporated interaction data other than direct physical bindings, such as protein phosphorylations, from a previously published paper [15] into our dataset. The final dataset was filtered to remove interactions from non-human sources, and the redundant and self-interacting pairs were excluded. In total, a human PPI network containing 22,616 nodes and 515,015 edges was constructed.

### 2.3. PPI Subnetworks among Proteins Encoded by Delirium-Associated Genes

We used the top-ranking genes with respect to the CTD inference score as seed genes and mapped them to the human PPI network, as described above, and then extracted the maximal connected component as the protein-interaction subnetwork, using the Cytoscape program (version 3.7.1) [19]. To identify highly interconnected PPI clusters, the resultant network was analyzed with the Molecular Complex Detection (MCODE) clustering tool [20], a plugin implemented in Cytoscape. The following parameters were applied: fluff density cutoff = 0.5, K-core = 2, node score cutoff = 0.2 and max depth up to 100. To further assess the associations between modules and delirium, Fisher’s exact test was used to determine whether differentially expressed genes (DEGs) for a mouse delirium model and delirium-associated genes derived from genome-wide association studies (GWASs) were significantly enriched in a target gene module. DEGs for the mouse delirium model were retrieved from a study by Xiang et al., who studied brain gene expression during perioperative neurocognitive disorders in mice [21]. Delirium-associated genes were retrieved from the Genome-Wide Association Studies Catalog (https://www.ebi.ac.uk/gwas/) [22] and the DisGeNET database [23]. Brain-expressed gene enrichment was determined by using the Enrichr web server [24]. The hub genes in the PPI networks were evaluated by using the Cytoscape-plugin CytoHubba [25]. In addition to the node degree, three of the most widely used parameters, i.e., betweenness centrality, maximal clique centrality (MCC), and maximum neighborhood component (MNC), were calculated to identify potential hub genes [25,26].

### 2.4. PPI Subnetworks Simulation

The connectivity of the delirium PPI network was quantified by the size of the largest connected component (LCC) and the shortest distance (SHD) between proteins [15]. Using the Python scripts revised from a previously published program, we calculated the LCC, SHD, and significance of each subnetwork [15]. To assess the significance of LCC and SHD, expected null distributions were calculated by randomizing sets of proteins of equal seed-list size. A total of 1000 random gene sets were generated. The *z*-score was calculated to estimate whether the two network parameters of the delirium PPI network were significantly different from the 1000 simulated networks.

### 2.5. Pathway and GO Enrichment Analysis

In order to investigate gene clusters at the molecular and functional level, we performed Reactome pathways, KEGG pathways, and GO (All) terms enrichment analysis by using clusterProfiler (http://bioconductor.org/packages/release/bioc/html/clusterProfiler.html) with a strict cut-off of FDR < 0.05 [27]. We implemented the compareCluster function in the clusterProfiler package, which calculates and compares enriched functional categories of each gene cluster. Using gene ratios (the proportions of genes enriched in each category) and adjusted *p*-values, we constructed a dot plot to visualize the difference in enriched functional categories between the clusters.

### 2.6. Psychiatric Disorder Association Analysis

Gene–disease association data were retrieved from the Psychiatric Disorders Gene Association NETwork (PsyGeNET) database [28], which contains information about psychiatric diseases and their associated genes that were designed to reveal genetic links to eight classes of psychiatric disorders. For these psychiatric disorder association analyses, the R psygenet2r package was used [29]. The enrichment of disease-associated genes was tested by using the hypergeometric distribution test. Hypergeometric *p*-values were calculated by using the phyper function in R package, and disorders with *p*-values less than 0.05 were considered to be significantly enriched.

### 2.7. Enrichment Analysis with Genes Located in HARs

HAR genes were retrieved from the study by Doan et al. [30], who identified them by comparative genome analysis, representing conserved genomic loci with elevated divergence in humans. A total number of 2737 human accelerated regions were identified, representing 2163 HAR-associated genes [30]. We also retrieved HAR-Brain genes, which were observed to be significantly more expressed in brain tissues and overlapped with the set of HAR genes compiled by Wei et al. [31]. There were 415 HAR-Brain genes. We used only HARs overlapped with protein-coding genes. Enrichment analysis was performed for the sets of HAR genes by means of the one-sided Fisher’s exact test implemented in the fisher.test function in R.

## 3. Results

### 3.1. PPI Network Analysis of Delirium-Associated Genes Derived from Chemical–Gene Interactions in the CTD

**Figure 1:**
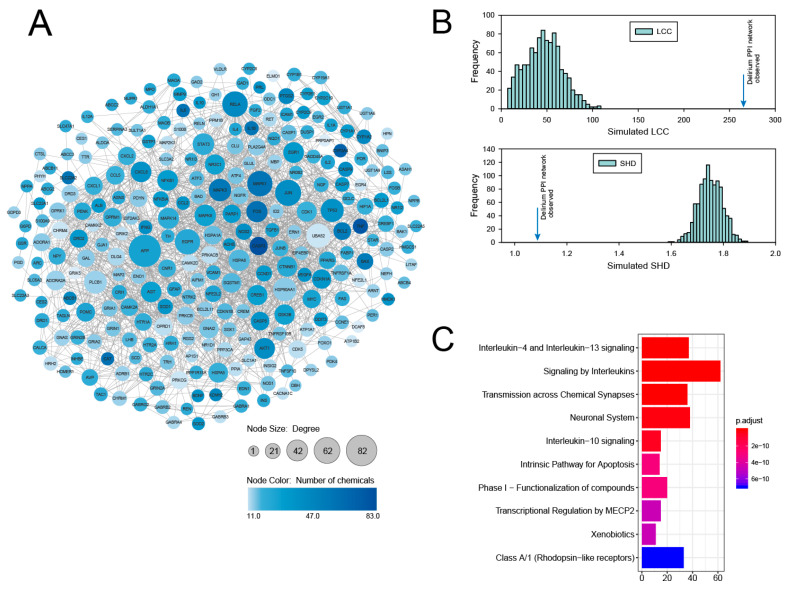
A delirium protein–protein interaction (PPI) network constructed with delirium-associated genes derived from the Comparative Toxicogenomics Database (CTD). (**A**) The delirium PPI network. Nodes represent delirium-associated genes and the node size is proportional to its degree. Node color (from lighter to darker) is proportional to the number of chemicals supporting chemical–gene interactions associated with delirium. (**B**) Largest connected component (LCC) size and shortest distance (SHD) measure of the delirium PPI network (blue arrows) in comparison to the expected, random distribution (histograms). The simulation results of 1000 random networks are shown in the histograms. (**C**) Functional enrichment analysis results of the delirium PPI network genes. Reactome pathway terms were determined by using the R package clusterProfiler. The top ten Reactome pathway terms are illustrated as bar plots, with the gene count denoted by the length of the bar, and significance denoted by color. The *p*-values were adjusted by the Benjamini–Hochberg method.

The CTD integrates genes and chemicals through the manual curation of chemical–gene, chemical–disease, and gene–disease relationships from diverse sources [13]. Using chemical–gene interactions associated with delirium in the CTD, we identified a total of 3573 genes that were associated with delirium with CTD inference scores > 20. After sorting this gene list by the CTD inference score, the 297 top-ranking genes with CTD inference scores > 40 were taken as the seed genes for further PPI analysis (Supplementary Materials Table S1). We investigated the PPI subnetworks among proteins encoded by the 297 top-ranking delirium-associated genes by using the human molecular interaction network. As a molecular interaction data source, we compiled the human PPI network from several datasets containing physical binary interactions among molecular components (see Materials and Methods). We mapped the 297 genes as seed genes to the human PPI network and obtained a delirium PPI network consisting of 266 nodes and 1709 edges (Figure 1A). This sub-network comprised highly interconnected nodes referred to as node degrees, as calculated by the Cytoscape plugin NetworkAnalyzer. As shown from the node color in Figure 1A, many nodes having higher degrees also exhibited a higher number of chemicals supporting the chemical–gene interactions associated with delirium.

To assess whether the proteins of the delirium PPI network interacted significantly or were due to a random chance effect, we generated 1000 random PPI networks and compared the size of the largest connected component (LCC) with the observed PPI network [15]. As shown in Figure 1B, the LCC of the delirium PPI network was significantly larger than expected by random chance (LCC = 266, *z*-score = 10.9). In addition to the size of the LCC, we also compared the shortest distance (SHD) of the network encoded by delirium-associated genes and random genes. We found that the SHD value of the delirium PPI network was shifted towards 1.0 and the observed value was significantly smaller compared with that of the random genes (SHD = 1.08, *z*-score = −14.5) (Figure 1B). Taken together, the significant network measurements demonstrate that the protein products of the delirium-associated genes derived from chemical–gene information in the CTD exhibit a high tendency to interact and agglomerate.

To explore the functional features of the delirium PPI network, we performed a Reactome pathway analysis, using clusterProfiler. The most significantly enriched Reactome pathways of the network are presented in Figure 1C. These pathways include signaling by interleukins, transmission across chemical synapses, intrinsic pathway for apoptosis, and xenobiotics. Moreover, the Reactome pathway analysis showed a total of 189 significant pathways, which were mainly related to neuronal processes, drug responses, and inflammation (Supplementary Materials Table S2). These pathways are consistent with previous reports for delirium that suggested the importance of neuroendocrine and inflammatory mechanisms [7].

### 3.2. Identification and Analysis of Densely Connected Modules in the Delirium PPI Network

**Figure 2:**
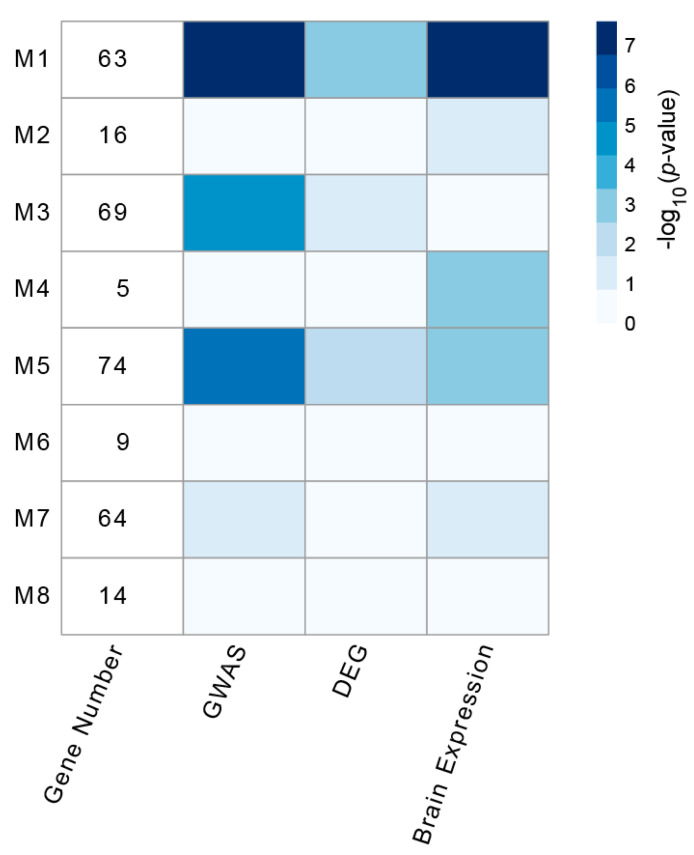
Densely connected modules in the delirium PPI network. Eight significantly connected PPI modules were extracted from the delirium PPI network, using the Molecular Complex Detection (MCODE) plugin. Module-level enrichment was assessed for three delirium-related gene sets: delirium-associated genes derived from genome-wide association studies (GWASs), differentially expressed genes for the mouse delirium model (DEG), and the brain-expressed gene set (Brain Expression). The blue colors in each cell indicate the *p*-value for significance of overlap, using Fisher’s exact test (−log_10_ (*p*-value)).

Disease-associated genes have a tendency to interact with each other and work together in the same biological module on the molecular interaction network [32]. The detection of such interaction modules could help identify the key pathways and genes for diseases and assist in revealing the molecular mechanisms underlying their etiology. To investigate whether delirium-associated genes may form a highly connected molecular module, we used the MCODE plugin [20] with tuned settings, instead of default settings, to identify pathway-like modules in the delirium PPI network (see Materials and Methods). As shown in Figure 2, MCODE analysis identified eight densely connected modules, with the members ranging from 5 to 74 genes. In order to prioritize the modules by their relevance to delirium pathogenesis, we next tested the modules for enrichment of DEGs in the mouse delirium model and delirium-related GWAS signals. Of the eight PPI modules, four modules (M1, M3, M5, and M7) were significantly enriched for the GWAS signals, while three modules (M1, M3, and M5) were significantly enriched for the DEG set (Figure 2). By also considering the brain-expressed gene set, we selected modules M1 and M5 as the top two delirium-relevant network modules; both of these modules were significantly enriched with all three gene sets used in this analysis (Figure 2).

**Figure 3:**
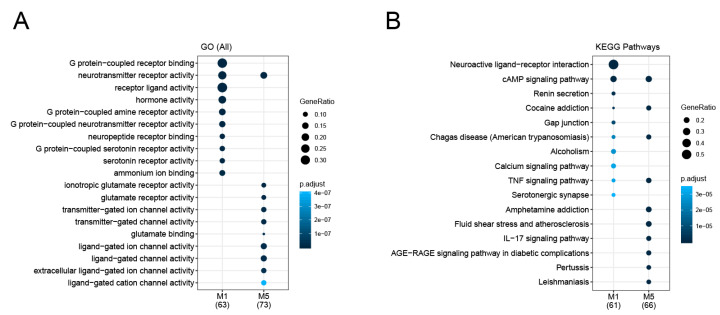
Gene Ontology (GO) and KEGG pathway enrichment analysis of the genes in the M1 and M5 modules. (**A**) GO enrichment analysis of the M1 and M5 module gene set. GO terms were determined for each gene set, using the compareCluster function in the R package clusterProfiler. The most over-represented GO terms are illustrated as dot plots, with the gene ratio denoted by size and the significance denoted by color. The *p*-values were adjusted by the Benjamini–Hochberg method. (**B**) KEGG pathway enrichment analysis of the M1 and M5 module gene set. Pathways were determined for each gene set using the compareCluster function in the R package clusterProfiler. The most over-represented pathways are illustrated as dot plots, with the gene ratio denoted by size and the significance denoted by color. The *p*-values were adjusted by the Benjamini–Hochberg method.

In order to elucidate the specific biological relevance of the M1 and M5 modules, 63 genes in the M1 module and 74 genes in the M5 module were subjected to Gene Ontology (GO) and KEGG pathway enrichment analyses. We found that module M1 was significantly associated with G protein signaling−related GO terms such as G protein−coupled amine receptor and G protein−coupled serotonin receptor (Figure 3A). For the M5 module, the significant GO terms were mainly ligand-gated ion channel activity and glutamate receptor activity. In addition, genes involved in neurotransmitter receptor activity were enriched in both modules. KEGG analysis showed that the two most significantly overrepresented pathways for the genes of the M1 module were neuroactive ligand−receptor interaction and the cAMP signaling pathway (Figure 3B). We confirmed four significantly enriched pathways common to both the M1 and M5 modules, which were the pathways for cAMP signaling, cocaine addiction, Chagas disease, and TNF signaling. Specific pathways for the M5 module genes, such as the pathways for amphetamine addiction and IL−17 signaling, were also evident. Collectively, these pathways and GO terms are mostly critical for neuronal response and function [33] and have frequently been linked to pathophysiological activities related to various psychiatric disorders [34,35]. These results indicate that the identified M1 and M5 modules are potentially important for delirium studies, and we focused on these modules in our subsequent studies. Full lists of the significantly enriched GO terms and KEGG pathways, as well as their associated genes, are provided in Supplementary Materials Table S3.

### 3.3. Psychiatric Disease Association of Delirium-Related Modules

**Figure 4:**
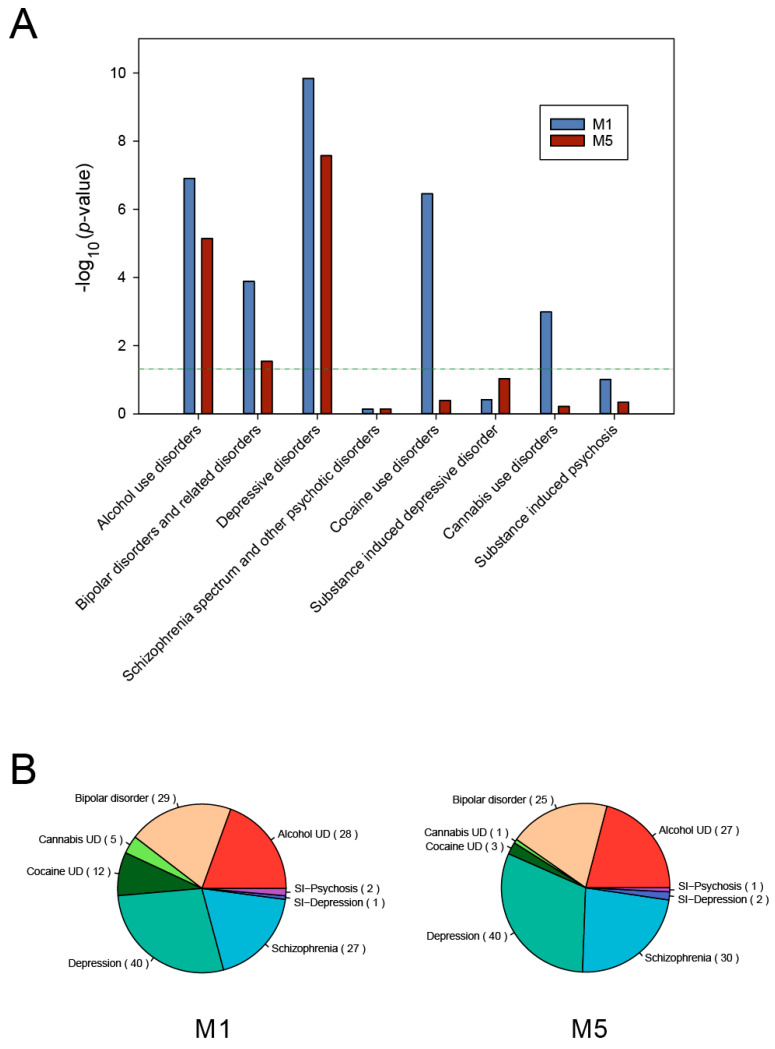
Psychiatric disease association of the delirium-related M1 and M5 modules. (**A**) Disease association analysis for the eight classes of psychiatric disorders. The analysis was performed by using the R psygenet2r package. The enrichment of disease-associated genes was tested by using the hypergeometric distribution test, and results with *p*-values less than 0.05 were considered as statistically significant. The dashed line indicates *p* = 0.05. (**B**) The distribution of genes associated with PsyGeNET disease categories between the M1 and M5 modules. The number in parentheses represents the associated genes in each module (SI, substance induced; UD, use disorders).

It has often been observed that risk genes in the different brain disorders are highly related and interconnected in the PPI networks [36]. In order to consider the associations of the delirium-related modules with multiple psychiatric disorders, we further examined whether a significant number of disease-associated genes were present in the M1 and M5 modules by using PsyGeNET, a platform designed to reveal genetic links to eight classes of psychiatric disorders [28]. We found that both the M1 and M5 module gene sets were significantly associated with several classes of psychiatric disorders. As shown in Figure 4A, we confirmed that five major classes of psychiatric disorders were significantly associated with the M1 module, which were alcohol-use disorders, bipolar and related disorders, depressive disorders, cocaine-use disorders, and cannabis-use disorders. In contrast, only three classes of disorders—including alcohol-use disorders and depressive disorders—were significantly associated with the M5 module. The distributions of genes associated with PsyGeNET disease categories in the M1 and M5 modules were mostly similar, but the genes associated with cannabis-use disorders and cocaine-use disorders were increased in the M1 module (Figure 4B).

### 3.4. Enrichment of HAR-Brain Genes in Delirium-Related Modules

**Figure 5:**
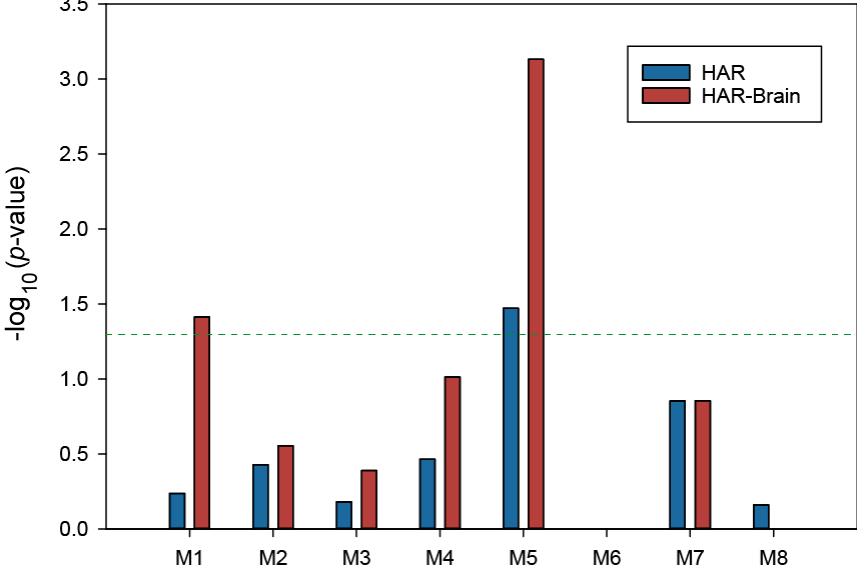
Enrichment of human accelerated region (HAR) and HAR-Brain genes in the delirium-related modules. Overlaps of module genes with HAR and HAR-Brain genes were examined, and the enrichment was tested by using Fisher’s exact test. Results with *p*-values less than 0.05 were considered statistically significant. The dashed line indicates *p* = 0.05.

**Figure 6:**
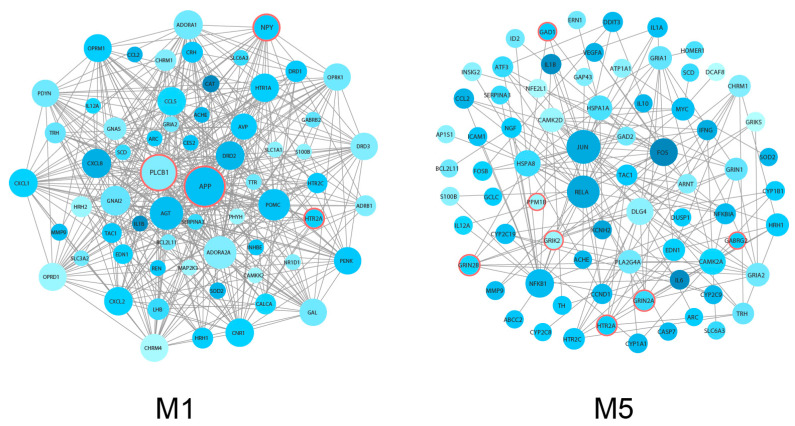
Top two delirium-relevant PPI modules, M1 and M5, prioritized by enrichment analysis. Nodes represent delirium-associated genes, and the node size is proportional to its degree. Node color (from lighter to darker) is proportional to the number of chemicals supporting chemical–gene interactions associated with delirium. The circles with thick red borders represent HAR-Brain genes.

The insights obtained from comparative evolutionary analysis of human genes have proven their value for the discovery of key disease genes [37]. In particular, genes associated with HARs identified by comparative genomic studies have been linked to neural development and also to neurological disorders, such as autism spectrum disorder [30]. Therefore, we performed a search in each module, using the HAR genes and HAR-Brain genes retrieved from the previous studies [30,31]. We then examined the enrichment of the modules with these HAR gene sets. As can be seen from the –log_10_ (*p*-value) in Figure 5, the delirium-associated M1 and M5 modules showed a strong tendency to contain HAR-Brain genes (Figure 5, brown bar). We observed statistically significant enrichment of the HAR-Brain gene set in the M1 and M5 modules, but significant enrichment for the HAR gene set only in the M5 module (Fisher’s exact test, *p* < 0.05). The identified HAR-Brain genes were APP, PLCB1, NPY, and HTR2A in the M1 module, and GRIN2A, GRIN2B, GRIK2, GAD1, PPM1B, GABRG2, and HTR2A in the M5 module; these genes are represented by the circles outlined with a thick red border in Figure 6. Most of these HAR-Brain genes encode proteins related to neural function (Table 1) and were mainly involved in KEGG pathways, such as neuroactive ligand–receptor interaction and amphetamine addiction (Figure 3B and Supplementary Materials Table S3).

To explore the hub gene properties, we calculated the network features scores of each node in the M1 and M5 modules by CytoHubba [25]. As shown in Figure 6, the M1 module turned out to contain more highly connected nodes than the M5 module (Supplementary Materials Table S4). The HAR-Brain genes in the M1 module showed higher node degree values compared with the HAR-Brain genes in the M5 module (Table 1). In addition, other hub gene parameters, such as betweenness centrality and MCC values, were also extremely high in HAR-Brain genes of the M1 module, and APP was indicated as the most connected node in this module (Table 1 and Supplementary Materials Table S4). These results suggest that the pathways associated with M1 module genes might be critically affected by the defects of these HAR-Brain genes, which could be potentially regarded as key genes for the delirium-development mechanism.

## 4. Discussion

Delirium is a neurobehavioral syndrome caused by dysregulation of neuronal activity, and many factors and mechanisms have been proposed, in an attempt to explain its etiology. The most commonly described neuronal factors associated with delirium include deficiencies in acetylcholine and melatonin availability; excess levels of dopamine, norepinephrine, and glutamate release; and alterations in serotonin, histamine, and gamma-aminobutyric acid regulations [5]. It is unlikely that any one of these factors is fully capable of explaining the etiology of delirium; rather, many associated genes appear to act together to lead to the complex cognitive and behavioral changes characteristic of the disease. Hence, knowledge of the inter-connectivity of delirium-associated genes in functional pathways is important to fully understand the mechanism of delirium development. In the present study, we implemented a systems-level approach to link chemical–gene associations to gene–disease information and the PPI network, and generated a subnetwork that provides unique insights into the mechanisms underlying the complex etiology of delirium. Based on the chemical–gene interactions associated with delirium in the CTD [13], we first compiled the 297 top-ranking seed genes and constructed a delirium PPI network consisting of delirium-associated seed genes. Our simulation analysis demonstrated that member genes of the delirium PPI network were significantly more interconnected than would be expected by chance. Moreover, the Reactome pathway analysis indicated that the delirium PPI network was mainly enriched for genes involved in neuronal processes, drug responses, and inflammation (Figure 1C and Supplementary Materials Table S2). Thus, rather than being randomly distributed within the human protein interaction network, the delirium PPI network genes formed local connections to each other because they interact to perform related biological functions. This revealed that the identified delirium PPI network would be useful for investigating the molecular mechanisms of delirium development.

Since small functional modules which might contain proteins participating in similar biological processes would be helpful to deduce delirium functional pathways, we applied the MCODE plugin to extract densely connected protein clusters in the delirium PPI network. In total, we identified eight significant functional modules consisting of highly connected protein members. Based on the significant enrichment of the delirium-associated gene sets, the top two delirium-relevant network modules, M1 and M5, were obtained. We then performed GO and KEGG enrichment analyses for both modules. The common functions and pathways of the genes in each module were mainly linked to neuronal functions and aberrations, indicating that these modules may potentially form biological complexes or pathways that are involved in delirium. To further explore the relations with multiple psychiatric disorders, we examined our module data, using PsyGeNET, a platform designed to reveal gene-level links to eight classes of psychiatric disorders [28]. The results indicated that there were significant numbers of neuropsychiatric-disorder-associated genes in the M1 and M5 modules, but the classes of associated psychiatric disorders were quite distinct between these two modules. Interestingly, while the GO terms such as G protein−coupled amine receptor activity and G protein−coupled serotonin receptor activity were specifically enriched with M1 module genes, the GO terms such as ligand-gated ion channel activity and ionotropic glutamate receptor activity were specifically enriched with M5 module genes (Figure 3A). These metabotropic and ionotropic receptors have been reported to be essential for various aspects of the nervous system functions [38], many of which have been associated with the pathophysiology of various psychiatric disorders [39,40]. Thus, our study provides mechanistic insights indicating the important role of neuronal receptor dysregulation in the pathophysiology of delirium, and the separate functional characterization of the two modules may provide information regarding the different aspects of delirium mechanisms associated with the M1 and M5 modules.

The divergence of HARs between humans and other species has been suggested to reflect potential roles in the evolution of human-specific traits, such as cognitive ability [30]. Evolutionary pressure on HARs regulating higher-order brain functions may also have been accompanied by an increased risk of psychiatric disorders [31,41]. Our present analyses provided evidence that HAR-Brain genes were significantly overrepresented in the delirium-relevant network modules M1 and M5. The HAR-Brain genes found in both modules directly relate to the signal transduction and development of the human central nervous system. For example, APP in the M1 module is involved in neural growth and maturation during brain development [42], and it plays a critical role in the pathology of Alzheimer’s disease [43]. PLCB1 and NPY in the M1 module are important for G-protein-coupled receptor signaling in neurons, together with HTR2A, whose full name is 5-hydroxytryptamine receptor 2A. GRIN2A, GRIN2B, and GRIK2 in the M5 module are glutamate ionotropic receptor subunits and believed to be essential in the central nervous system. GAD1 and GABRG2 in the M5 module are known as gamma-aminobutyric acid (GABA) synthesis enzyme and GABA type A receptor subunit gamma2, and are essential in regulating neurotransmitter release and neuronal excitability. These results are consistent with previous investigations which demonstrated that HAR-Brain genes function as important neuronal regulators and key players in neurogenesis [31]. It is very likely that the HAR-Brain genes identified in both modules specifically increase the risk of delirium and related neuronal disorders through the dysregulation of the module-associated pathways. Most importantly, the HAR-Brain genes in the M1 module are all reported to be involved in the pathology of Alzheimer’s disease [43,44,45,46], which is frequently characterized by delirium symptoms [47]. Moreover, the present study identified APP, which plays key roles during Alzheimer’s disease progression [43], as the most highly connected hub gene of the M1 module. Therefore, it is possible that Alzheimer’s disease pathogenesis and delirium development share common pathways and important key risk genes. The misregulation of the four HAR-Brain genes, namely APP, PLCB1, NPY, and HTR2A, in the M1 module is more likely to underlie delirium susceptibility rather than specifically be the molecular pathways driving the development of delirium. Further analysis of the HAR-Brain genes in both modules may provide new insights into the molecular mechanisms of delirium development and pinpoint clues for delirium treatment.

In conclusion, we carried out system-level analysis of delirium-associated genes derived from chemical–gene interactions in the CTD and detailed the PPI network characteristics of delirium-associated genes. Our simulation results showed that delirium-associated genes formed a highly connected PPI network, indicating a high tendency to interact and agglomerate. We identified two densely linked modules, M1 and M5, that were significantly associated with delirium and showed that genes related to neurotransmitter receptor activity were enriched in both modules. Moreover, our analyses provided evidence that the HAR-Brain genes were overrepresented in the delirium-relevant network modules, M1 and M5. In particular, the four HAR-Brain genes, i.e., APP, PLCB1, NPY, and HTR2A, in the M1 module appear to exhibit hub properties and thus might be used as therapeutic intervention targets and diagnostic biomarkers. However, potential biases from manual curation techniques in the CTD could shift the findings in this study. Therefore, genetic/molecular associations with delirium found here need to be confirmed in another study with rigorous quantitation of delirium outcomes and assessment of other risk factors, such as age, Alzheimer’s disease, and inciting insult leading to delirium. Additional experimental studies are also required to verify these key target genes and molecular pathways.

## Figures and Tables

**Table 1:** HAR-Brain genes found in the M1 and M5 modules.

Symbol	Gene Full Name	Module	Degree	Betweenness	MCC
*APP*	amyloid beta precursor protein	M1	45	1862.02222	9.22 × 10^13^
*PLCB1*	phospholipase C beta 1	M1	37	353.11032	9.22 × 10^13^
*NPY*	neuropeptide Y	M1	21	0.58095	9.22 × 10^13^
*HTR2A*	5-hydroxytryptamine receptor 2A	M1, M5	10	12.21667	15,144
*GRIN2A*	glutamate ionotropic receptor NMDA type subunit 2A	M5	7	0	5040
*GRIN2B*	glutamate ionotropic receptor NMDA type subunit 2B	M5	7	0	5040
*GRIK2*	glutamate ionotropic receptor kainate type subunit 2	M5	4	1.33333	12
*GAD1*	glutamate decarboxylase	M5	3	36.12047	4
*PPM1B*	protein phosphatase, Mg^2+^/Mn^2+^ dependent 1B	M5	1	0	1
*GABRG2*	gamma-aminobutyric acid type A receptor subunit gamma2	M5	1	0	1

MCC = maximal clique centrality.

## Supplementary Materials

The following are available online at https://www.mdpi.com/2073-4425/11/10/1225/s1. Table S1: The top-ranking delirium-associated genes with CTD inference scores > 40 used in the present study. Table S2: Reactome pathway enrichment results of the delirium PPI network genes. Table S3: GO and KEGG pathway enrichment results of the M1 and M5 module genes. Table S4: The network features scores of each node in the M1 and M5 modules analyzed by the CytoHubba program.
